# Normal Roles for Dietary Fructose in Carbohydrate Metabolism

**DOI:** 10.3390/nu6083117

**Published:** 2014-08-05

**Authors:** Maren R. Laughlin

**Affiliations:** Division of Diabetes, Endocrine and Metabolic Diseases, National Institute of Diabetes and Digestive and Kidney Diseases, National Institutes of Health, 6707 Democracy Blvd, Room 787, MSC 5460, Bethesda, MD 20892, USA; E-Mail: maren.laughlin@nih.gov; Tel.: +1-301-594-8802

**Keywords:** fructose, carbohydrate, metabolism, liver

## Abstract

Although there are many well-documented metabolic effects linked to the fructose component of a very high sugar diet, a healthy diet is also likely to contain appreciable fructose, even if confined to that found in fruits and vegetables. These normal levels of fructose are metabolized in specialized pathways that synergize with glucose at several metabolic steps. Glucose potentiates fructose absorption from the gut, while fructose catalyzes glucose uptake and storage in the liver. Fructose accelerates carbohydrate oxidation after a meal. In addition, emerging evidence suggests that fructose may also play a role in the secretion of insulin and GLP-1, and in the maturation of preadipocytes to increase fat storage capacity. Therefore, fructose undergoing its normal metabolism has the interesting property of potentiating the disposal of a dietary carbohydrate load through several routes.

## 1. Introduction

There have been a considerable number of recent studies designed to understand whether fructose, in the current western diet rich in fructose-containing sugars, contributes uniquely to obesity, diabetes, and their complications. Fructose is, however, an important nutrient found naturally in fruit as a component of most healthy diets and it may also be of interest to review the roles played by this sugar in normal metabolism. Much of the recent literature, while providing important health information, is not relevant for the purpose of understanding the metabolism of low levels of fructose eaten as a minor fraction of the carbohydrate found in a well-balanced meal. Experimental subjects are often exposed to substantial doses of a single monosaccharide to exaggerate any effects so that they can be detected in a reasonable time, while fructose is rarely consumed in isolation under normal circumstances. Glucose derived from the same meal is metabolized in synergy with fructose. Therefore, only studies where fructose and glucose are consumed together can yield insight regarding the normal metabolic fate of the sugars.

Sugar absorption is a simple example of the synergy of fructose metabolism with that of glucose. When eaten alone or in great excess, especially relative to glucose, many people experience fructose intolerance due to inability to absorb fructose from the gut into the bloodstream. However, fructose is easily absorbed in the great majority of people when eaten as part of the disaccharide sucrose, or together with glucose at roughly similar or higher levels [1].

In the current paper, normal fructose metabolism will be discussed, including several ways in which it synergizes with glucose to increase the rate of disposal of a dietary carbohydrate load.

## 2. Discussion

### 2.1. Fructose Transporters and Concentrations in the Blood

The normal roles of fructose in human metabolism are circumscribed by the amounts of fructose (and diet-derived glucose) found in a meal, the time course of absorption from the gut, and the concentrations of these sugars and their metabolic intermediates found in different parts of the bloodstream throughout the body. These parameters together with the sugar transporters, receptors and metabolic enzymes in tissues govern the fate of dietary fructose. Fructose enters the bloodstream more slowly than glucose and its levels are much lower, but they persist longer in the circulation. After healthy adults ingested 24 oz. of soft drink containing 69 g sucrose (half of which, 34.5 g, is fructose), fructose concentration in the peripheral venous blood rose >60-fold from a fasting level of about 0.005 mM to a maximum of 0.317 mM, and returned toward baseline levels by about 3 h after ingestion. Meanwhile, glucose rose from about 5.5 mM to 6.8 mM and returned to baseline by 90 min, accompanied by insulin release that peaked at about 30 min. Lactate, a major byproduct of hepatic fructose and glucose anaerobic metabolism, rose from 0.7 to 2 mg/dL at 60 min, and returned to baseline by 3 h [2]. As a point of reference, a 12 oz. soda contains about 17–22 g fructose, while a typical apple, pear or cup of grapes would contain about 12 g fructose [1]. The typical western diet is rich in sugars and contains on average 49 g fructose/day of which only 8 g are from natural sources such as fruits [3]. Forty-one grams of added fructose is the amount found in approximately 82 g sucrose (313 kcal). This is well above the AHA recommendation for a healthy diet, which limits added sugars to 100 kcal/day (26.2 g/day) for women and 150 kcal/day (39.3 g/day) for men [4], or the Health and Human Services/USDA Dietary Guidelines for Americans 2010, which, rather than addressing dietary sugars in isolation, recommend a combined limit for solid fats and added sugars of 5%–15% of total calories [5].

The liver is the major site for fructose metabolism. Fructose enters the portal circulation from the gut, and there is typically a dramatic gradient of fructose between blood entering and leaving the liver, which can be measured in animal models. Therefore, the liver (and pancreas) is exposed to much higher fructose concentrations than other non-splanchnic organs such as brain, skeletal muscle and heart. When 2 g/kg sucrose was delivered directly into the stomach of fasted rats, there was little difference between fructose levels in arterial blood from the aorta and peripheral venous blood, which both rose from 0.02 mM to about 0.15 mM. However, fructose was much higher in the portal vein, where it rose from 0.1 mM at baseline to ~1 mM at 30 min, and persisted at 0.6 mM when measured 60 min after gavage. During the same time, portal glucose rose much more, from 12 mM to about 20 mM [6]. Similar time courses were seen in healthy male baboons given 2 g/kg sucrose by gavage, where portal vein fructose peaked at 1.9 mM at 60 min and peripheral blood peaked at 0.6 mM [7]. It is difficult to achieve much higher blood fructose levels than these as fructose or sucrose absorption from the gut may be rate limiting, although blood fructose levels and its metabolic effects can persist for considerable time after a meal [8,9]. Therefore, experiments that use sucrose or fructose plus glucose, and limit blood fructose concentrations to these concentration and time ranges, are likely to be most informative regarding normal fructose metabolism.

There are several sugar transporters that can use fructose as a substrate, but the main physiological carriers are thought to be GLUT2 and GLUT5. GLUT2 is a high capacity, low avidity transporter for both glucose (K_M_ = 17 mM) and fructose (K_M_ = 66 mM). Unlike the glucose transporter GLUT4, it is not sensitive to insulin. In addition to the intestinal epithelium where it participates in sugar absorption, it is found in kidney, and in liver and beta cells where it acts to sense blood glucose concentration as well as transport glucose and fructose [10]. GLUT5 appears to be highly specific for fructose, and has a wider distribution than would be predicted given the high K_M_ of 6–10 mM and the typically low post-prandial circulating concentrations of fructose. Like GLUT2, GLUT5 is mainly found in intestine where it participates in fructose absorption, but also in kidney, fat, sperm, testes, brain and skeletal muscle. The exact role of GLUT5 and fructose metabolism in tissues outside of the gut is incompletely understood [11]. As mentioned above, fructose is not absorbed from the gut very efficiently when eaten as the monosaccharide alone. This is likely due to the low activity of GLUT5 and GLUT2 toward fructose. Few people have issues though, when fructose is part of sucrose, or in a meal that contains glucose or starches that are converted to glucose. In fact, absorption of fructose is maximal when present in a 1:1 ratio with glucose. The mechanism is not clear as there doesn’t seem to be synergy between fructose and glucose at either GLUT2 or GLUT5. There is some evidence in rats that the brush-border disaccharidase-related transport system, which cleaves sucrose and transports the resultant sugar monomers, can also transport fructose and glucose when present together. When the protein is inhibited, the ability of glucose to potentiate fructose absorption is abolished [12]. It is not clear what fraction of free fructose enters the circulation via the disaccharidase-related transport system in a normal diet, or whether the kinetics of free fructose plus glucose absorption is different than that of sucrose.

In addition to a means of entry, a tissue that uses fructose must have the enzymes necessary for its metabolism. Liver contains a specialized set of three enzymes, ketohexokinase (KHK, fructokinase), aldolase B, and triokinase. Fructokinase phosphorylates fructose to fructose-1-phosphate which activates the sugar for further metabolism via aldolase B and triokinase into 3-carbon glycolytic intermediates [13,14,15]. There are two isoforms; KHK-C (K_M_ = 0.1 mM) is found in hepatocytes and the straight segment of the proximal renal tubule as well as pancreas and duodenum. KHK-A has lower affinity for fructose and is more widely distributed in pancreas, intestine, brain, lung, eye, adipose, spleen, skeletal muscle, heart, uterus, and adrenal [16]. The role of KHK-A in these extra-hepatic tissues is not entirely clear but it does appear to have metabolic impact. When both isoforms of fructokinase are absent, mice are protected from health effects of a high fructose diet. However, KHK-A-null mice have dramatically increased liver fructose levels and liver fat on a high fructose diet, implying that extrahepatic organs do metabolize fructose and reduce fructose uptake by the liver when the sugar is chronically elevated [17]. There may also be conditions under which fructose is phosphorylated directly to fructose-6-phosphate by hexokinase in some tissues, where it competes weakly with glucose as a substrate [18,19].

### 2.2. Fructose Promotes Carbohydrate Disposal through Its Actions in the Liver

It is well documented that the majority of ingested fructose is absorbed in its first pass through the liver (71% in fasted rats, 55% in the fed state). Metabolism in liver is responsive to blood fructose due to very high fructokinase activity, which allows almost free entry of fructose into the hepatic glycolytic and gluconeogenic pathways [9]. Whether eaten alone or with glucose, the vast majority of fructose is converted to trioses in the liver, which in turn are released into the bloodstream as lactate for oxidation in extrahepatic tissues, or converted to glucose via gluconeogenesis, which is released or stored as glycogen. The fraction stored depends on the need to maintain plasma glucose, and therefore glucose release is favored in the fasted state or during exercise, while glycogen storage is enhanced in the post-prandial state. A small fraction may be converted to lipid or oxidized in the liver [15,20,21].

Unlike that of fructose, the glucose economy of the body is tightly regulated, as glucose is an important nutrient for most tissues, but hypoglycemia and hyperglycemia both have health consequences. The liver is a major player in whole body glucose regulation, and a substantial portion of ingested glucose is taken up by liver during postprandial hyperinsulinemia and hyperglycemia [22]. There is considerable evidence that fructose facilitates this hepatic glucose disposal.

Cherrington and colleagues have explored this in some detail. They gave healthy and type 2 diabetic people an oral glucose tolerance test (OGTT) of 75 g glucose with or without 7.5 g fructose. Even though the addition of fructose increased the total sugar dose by 10%, plasma glucose was reduced by 19% in healthy people when fructose was included, and by 14% in those with diabetes. Although the insulin response was unaltered in healthy subjects, diabetics experienced a 21% reduction in plasma insulin when fructose was present. Venous fructose was doubled after fructose ingestion, but actual levels remained modest (0.04–0.06 mM) [23,24]. Similarly, a low dose of fructose infused intravenously into type 2 diabetic patients restored the ability of hyperglycemia to suppress hepatic glucose production [25]. Therefore, a small dose of fructose along with glucose has the effect of increasing glucose tolerance in even insulin-resistant people.

A large part of the means by which fructose increases glucose disposal is due to its powerful ability to catalyze liver carbohydrate storage. This was demonstrated in people using ^13^C nuclear magnetic resonance spectroscopy (MRS) during a hyperinsulinemic, euglycemic clamp. ^13^C-1-glucose, which is an MRS-visible version of normal glucose, was infused into healthy fasted people with or without the addition of 3.5 μol/kg/min unlabeled fructose, which had the effect of doubling venous plasma fructose to 0.28 mM. This is similar to blood fructose seen after ingestion of a high sugar meal [2]. Even though plasma glucose was kept constant at a basal level of 5 mM, hepatic glucose uptake was more than doubled by the presence of fructose, from 0.31 to 0.79 mmol/L/min. Liver glycogen made from the ^13^C-labeled glucose was monitored over time. Net liver glycogen synthesis increased almost four-fold from 0.14 to 0.54 mmol/L/min when fructose was added. The source of carbon for this extra glycogen was predominantly glucose, not fructose [26].

Cherrington and his colleagues conducted a series of experiments in the fasted conscience dog to more thoroughly explore the mechanisms whereby fructose exerts its effects on carbohydrate economy. Arterial glucose was clamped at 12.5 mM (hyperglycemia) and fructose was infused into the portal vein to achieve a series of concentrations reflective of the post-prandial state (maximum portal vein fructose was 0.43 mM). Liver fructose uptake was a function of its portal concentration. It peaked at 5 moL/kg/min, which represents extraction of about 50% of available fructose. At the same time, glucose uptake was dramatically increased from 14 μmol/kg/min without fructose, to 69 μmol/kg/min with fructose infusion [27]. Therefore, a small increase in portal fructose and hepatic fructose uptake catalyzed a more than 10-fold larger increase in glucose uptake at constant glucose and insulin.

It is more interesting to ask what occurs in the liver with ingested glucose and fructose. Again using dogs, the same group infused glucose into the duodenum (44 μmol/kg/min) with or without a small dose of fructose (2.22 μmol/kg/min), a protocol which might mimic a carbohydrate-rich meal. Although glucose absorption from the gut into the bloodstream was unaltered by the presence of fructose, hepatic glucose uptake was stimulated from 17 μmol/kg/min (48% of absorbed glucose) to 28 μmol/kg/min (70% of absorbed glucose). Hepatic fructose uptake itself was only 1.4 μmol/kg/min (84% of absorbed fructose). Once again, fructose uptake catalyzed an order of magnitude higher glucose uptake. Much of this additional glucose was stored as glycogen, and a substantial fraction was converted to lactate for use in extrahepatic tissues (total lactate appearance was 1.4 mmol/kg, which increased to 3.3 mmol/kg with fructose) [22].

These large catalytic effects on canine liver glucose metabolism are accomplished at fairly low levels of fructose—after glucose and fructose ingestion the portal vein fructose concentration rose 10-fold, but at 0.1 mM it was still only a few per cent of portal glucose concentrations. It is not clear how these small levels of fructose compete with the much higher levels of glucose for transport into liver through GLUT2. However, it is thought that the major fructose effect on glucose uptake is via direct effects of fructose-1-phosphate on glucokinase activity, the hepatic isoform of hexokinase, which catalyzes the transfer of a phosphate group from ATP to glucose to form glucose-6-phosphate [13,28,29]. The concentration of fructose-1-phosphate in the liver is highly labile and responsive to blood fructose, and can achieve considerable concentrations [8]. The inactive form of glucokinase is retained in the nucleus bound to glucokinase regulatory protein (GKRP). Fructose-6-phosphate, a glycolytic intermediate, is an allosteric repressor that helps maintain GKRP binding, while fructose-1-phosphate displaces fructose-6-phosphate, leading to activation and translocation of glucokinase to the cytosol. The glucose-6-phosphate produced by activated glucokinase plays a variety of regulatory roles, and in particular activates glycogen synthase. This is likely the mechanism whereby liver glycogen synthase activity and glycogen synthesis are increased following fructose gavage in rats [8]. It is interesting to note that were fructose phosphorylated directly to fructose-6-phosphate by hexokinase, it would not stimulate glucose uptake and storage. The two specialized sugar kinases, fructokinase and glucokinase, therefore orchestrate the observed synergy between glucose and fructose metabolism in liver.

There is considerable literature showing detrimental effects of fructose on liver high energy phosphates and lipid metabolism in people and animals [30]. Whereas lipid metabolism is outside the scope of this discussion, a few words regarding liver energy status are warranted. Intravenous infusion of fructose solution in animals or people can result in a dramatic loss of the high energy compound ATP due to very quick transfer of its phosphate group to form fructose-1-phosphate, and this change in energy status can impact metabolism [30,31]. However, severe ATP depletion is likely an artifact of fructose infusion. Hepatic ATP loss is minimal and transient after rats ate substantial fructose, enough to raise fructose-1-phosphate from 0.1 to 3.3 μmol/g wet wt [8,32]. Therefore, under normal circumstances in healthy animals and people, gut transport slows the appearance of fructose in portal blood and limits the rate of fructose phosphorylation, which might otherwise alter liver high energy phosphate concentrations or energy homeostasis.

### 2.3. Does the Pancreatic Islet Respond to Fructose?

As noted above, small doses of fructose along with glucose stimulate glucose clearance and reduce plasma glucose and insulin concentrations. However, large doses of fructose appear to both autonomously stimulate insulin secretion and augment glucose-stimulated insulin secretion (GSIS) *in vivo*. After people consumed 75 g of fructose, they experienced a modest elevation in blood glucose, GLP-1 and insulin, all of which were much smaller than that observed after a similar glucose meal [33]. However, when people ingested fructose after a glucose meal, when plasma glucose was already elevated, the fructose appeared to augment insulin secretion without a further substantial increase in blood glucose [34]. This fructose-sensitive insulin response was also seen during intravenous fructose infusion in people, and again, insulin release in response to hyperglycemia was increased by fructose [35].

This is at least partly due to direct effects of fructose on the pancreatic islet beta cell, since high concentrations of fructose (10–30 mM) elicit insulin secretion from isolated human and rodent islets. Lower levels of fructose that are similar to those found in the portal circulation after fructose ingestion (3–5 mM) are able to potentiate GSIS under hyperglycemic conditions. GSIS is dependent on uptake and metabolism of glucose in the beta cell. These cells express GLUT2 and are able to transport fructose, however fructose uptake and metabolism can be an order of magnitude lower than glucose usage at the same concentrations. This might explain the poor secretagogue action of fructose at low levels of glucose. Glucose and fructose uptake into the beta cell are dependent only on the concentration of each sugar, and one does not influence the uptake of other. Therefore sugar metabolism cannot explain the observed synergy between fructose and glucose with regard to insulin secretion [36,37].

Recently, the role of taste receptors in insulin secretion has been explored. Beta cells express T1R2 and T1R3, the subunits that together make up the sweet taste receptor heterodimer. In addition to fructose, the non-nutritive sweeteners sucralose, saccharin and acesulfame K elicit insulin secretion and potentiate GSIS at basal (3 mM) and elevated glucose (25 mM) in MIN6 cells, as does sucralose from mouse islets and saccharin in human islets [36,38]. Tyrberg and colleagues showed that an intravenous dose of 1 g/kg fructose was able to elicit an insulin response prior to measurable hyperglycemia in wild type mice. A smaller dose (0.3 g/kg) that was unable to elicit an insulin response at euglycemia, doubled the insulin response when 0.5 g/kg glucose was infused simultaneously. These fructose responses were completely abolished in mice lacking the T1R2 receptor, despite the fact that T1R2-null mice mounted a normal insulin response to glucose infusion or OGTT. Similar observations were made in isolated mouse islets from wild type and T1R2-null mice, and in human islets, where-fructose potentiated GSIS was abolished when T1R3 was inhibited with lactisole. Fructose exposure caused a large increase in intracellular calcium concentration above that seen with glucose, and phospholipase C inhibition abolished both fructose-associated calcium changes and potentiation of GSIS [36]. These experiments indicate that at the levels of fructose that are likely to be obtained in the portal circulation after a fructose-rich meal, fructose can substantially augment GSIS via a mechanism that involves the beta cell sweet taste receptor.

There is some evidence that fructose may also affect GSIS indirectly, through the action of GLP-1. GLP-1 is released from enteroendocrine L cells after a meal in response to luminal glucose and has been a subject of considerable recent interest, as it regulates gut motility, modulates appetite through receptors in the CNS, and potentiates pancreatic islet beta cell insulin secretion [39]. GLP-1 is also released in response to large fructose loads, and so it is reasonable to ask whether this is another mechanism whereby fructose can stimulate insulin secretion. In a recent study, 75 g of ingested fructose potentiated plasma GLP-1 in healthy people, but no rise in GIP, PYY or CCK was observed. The GLP-1 response was substantially smaller than that seen with a comparable glucose meal. Fructose potentiation of GSIS was not accompanied by an additional rise in GLP-1 and therefore unlikely to be GLP-1 dependent. This experiment did not test whether synergy exists between the two sugars at the L cell in the gut, as the two sugars were not consumed at the same time. In another study in normal human subjects, post-prandial GLP-1 secretion was augmented when a fructose drink was part of the meal relative to a glucose drink, but the study was not designed to test whether GLP-1 affected insulin secretion [40]. The GLP-1 specific response to oral fructose was also seen in rats and mice, and in GLUTag cells in response to fructose in the culture media, where it seems to involve K_ATP_ channel activity [33,34]. Similarly to pancreatic beta cells, L cells can secrete GLP-1 in response to sweet non-nutritive compounds, which is inhibited by lactisol. It is therefore possible that the taste receptor is involved in fructose’s role as a GLP-1 secretagogue [41]. It remains to be seen if there are conditions under which fructose-induced GLP-1 is physiologically meaningful, whether through potentiation of insulin secretion or its other roles in gut and brain.

### 2.4. Fructose Oxidation

The ultimate endpoint of fructose metabolism is oxidation in extrahepatic tissues in the form of glucose, lactate or fatty acids manufactured in and released from the liver. In another instance in which fructose plays a catalytic role, small amounts of fructose ingested along with glucose serve to increase the rate of oxidation of the exogenous carbohydrate load. In people during cycling exercise, combustion of ingested carbohydrate was 55% higher when fructose was present with glucose in a 1:2 ratio *vs.* glucose alone [42]. Fructose contributes substrate for gluconeogenesis and quickly appears in the blood as glucose after people eat a meal [21,43]. The presence of fructose can increase the overall rate of glucose appearance slightly, but as plasma glucose is tightly controlled the net effect of fructose-induced gluconeogenesis is more often a higher rate of hepatic glycogen storage than an increased rate of glucose appearance in the bloodstream for extrahepatic oxidation [26,43]. On the other hand, even small doses of fructose elevate plasma lactate considerably above that of glucose alone, and extrahepatic lactate oxidation is largely a function of plasma lactate concentration. During cycling exercise, the inclusion of fructose ingested with glucose raised circulating lactate appearance and oxidation by 30% relative to glucose alone, and this was equal to the excess exogenous carbohydrate oxidation potentiated by fructose [43]. Thus, oxidation of lactate in heart, skeletal muscle and other organs serves as a means by which fructose increases the rate of disposal of a carbohydrate load and thereby spares glycogen stores.

Since extrahepatic fructose disposal is predominantly via oxidation of lactate and glucose derived from fructose in the liver, it is therefore somewhat surprising that fructose-specific GLUT5 is the second most abundant sugar transporter in human skeletal muscle, with expression levels of about a third of that of the major transporter GLUT4. Interestingly, Type I oxidative red fibers tend to be rich in GLUT4 and GLUT12 (the third most abundant sugar transporter), while GLUT5 is most abundant in Type IIb white fast twitch glycolytic fibers [44]. GLUT5 is elevated in both fiber types in Type 2 diabetic subjects, and normalized by treatment with pioglitazone [45]. Exercise training in sedentary adults had the expected effect of increasing GLUT4 by 66% in skeletal muscle, but it also caused a dramatic 72% reduction of GLUT5 [46]. Taken together, it appears as though sedentary behavior and the insulin resistance of type 2 diabetes may be accompanied by elevated GLUT5 as well as depressed GLUT4 expression, and this pattern is reversed by exercise or drug treatments aimed at improving skeletal muscle insulin resistance.

Skeletal muscle has the ability to metabolize considerable fructose when plasma fructose is highly elevated, although it is not clear whether fructose is oxidized directly in muscle at the plasma levels found after a fructose-rich meal. Young healthy fasted men exercised on a bicycle during continuous infusion of fructose that raised arterial blood levels to 5.5 mM, which is an order of magnitude higher than typical post-prandial concentrations. Arterial-venous differences across the splanchnic and leg muscle beds showed that whole body muscle uptake of fructose during exercise (4.7 mmol/min) exceeded splanchnic uptake (3.8 mmol/min). During recovery from exercise, muscle fructose uptake remained comparable to splanchnic utilization. Muscle glucose uptake during exercise was unaffected by the presence of fructose [47].

These data show that human skeletal muscle can directly metabolize fructose when plasma levels and energy demand are both high. They show that the fructose metabolic machinery is induced in states that are typically insulin resistant. They do not address whether there are any normal circumstances under which muscle participates in the clearance of plasma fructose per se following a fructose-rich meal, and if so, whether muscle fructose metabolism is altered by exercise, sedentary behavior or metabolic disease.

### 2.5. Fructose May Facilitate Fat Storage through Adipocyte Maturation

Adipose tissue stores excess dietary nutrient as triglyceride, and maintenance of sufficient mature adipocytes for this purpose is imperative for the health of other tissues. These cells are highly insulin responsive and take up considerable glucose. Fat cells in culture can also metabolize fructose, and a recent intriguing line of research explores a role for fructose in stimulation of preadipocyte maturation. Were this to occur *in vivo* under normal physiological conditions, it would be another way in which fructose facilitates the disposal of a carbohydrate-rich meal.

5.5 mM fructose as the sole nutrient is sufficient to support differentiation of murine 3T3-L1 fibroblasts into adipocytes. Low levels of fructose that mimic those found in the venous circulation following a fructose-rich meal (0.05–0.55 mM), when added to media containing 11.1 mM glucose also potentiate adipogenesis and increase lipid stores in 3T3-L1 cells. GLUT5 knockdown or inhibition severely reduces the catalytic effects of fructose, indicating that fructose transport into the cell is required [48]. Therefore, physiological levels of fructose and glucose together induce expansion of adipose tissue cells, and participate in disposal of a carbohydrate load through fat storage. In support of this idea, chow-fed GLUT5-null mice had less than half the white adipose tissue mass of wild type mice, and embryonic fibroblasts from the GLUT5-null mice resisted differentiation in culture [49]. Interestingly, the levels of GLUT5 in 3T3-L1 cells fell dramatically during differentiation, and GLUT5 was reduced with age in fat tissue from lean and obese Zucker rats, suggesting that the ability of adipose to import fructose may be altered with metabolic state [49,50].

Adipocytes and preadipocytes both contain T1R2 and T1R3, the components of the sweet taste receptor, and the non-nutritive sweeteners saccharin stimulates adipogenesis from 3T3-L1 cells, mouse ear mesenchymal stem cells, and human adipose stromal vascular cells. However, it does not appear that taste receptors are involved in the process, as saccharin is equally effective in differentiation of ear mesenchymal stem cells from wild-type, T1R2 or T1R3 knockout mice [51]. There is even some data showing that signaling through taste receptors suppresses maturation of 3T3-L1 cells [52]. Therefore, while evidence is emerging for a role of fructose in adipocyte maturation and lipid storage, more research is needed.

## 3. Conclusions

Fructose is part of a typical diet. Although there are health risks for sedentary people on a sugar-rich diet, there are also important metabolic roles for modest levels of fructose. Fructose enhances glucose metabolism, and as part of a healthy meal acts through several mechanisms to facilitate disposal of a dietary carbohydrate load.

There are many important questions that remain concerning roles for fructose in other aspects of normal human health. In particular, recent studies indicate that large doses of fructose given alone or as part of a meal may delay satiety, through endocrine signals and nutrient detection in the central nervous system, and thereby increase calorie intake [40,53]. Further research is needed to determine if fructose works through these pathways to increase body weight, and whether modest levels of fructose, particularly ingested in combination with glucose as part of a healthy meal, similarly impact eating behavior.
